# Distinct Impairments Characterizing Different ADNP Mutants Reveal Aberrant Cytoplasmic-Nuclear Crosstalk

**DOI:** 10.3390/cells11192994

**Published:** 2022-09-26

**Authors:** Maram Ganaiem, Gidon Karmon, Yanina Ivashko-Pachima, Illana Gozes

**Affiliations:** The Elton Laboratory for Molecular Neuroendocrinology, Department of Human Molecular Genetics and Biochemistry, Sackler Faculty of Medicine, Sagol School of Neuroscience and Adams Super Center for Brain Studies, Tel Aviv University, Tel Aviv 6997801, Israel

**Keywords:** activity-dependent neuroprotective protein (ADNP), ADNP syndrome, CRISPR/Cas9, green fluorescent protein (GFP), immunocytochemistry, live cell imaging, NAP (davunetide)

## Abstract

(1) Background: Activity-dependent neuroprotective protein (ADNP) is essential for neuronal structure and function. Multiple de novo pathological mutations in ADNP cause the autistic ADNP syndrome, and they have been further suggested to affect Alzheimer’s disease progression in a somatic form. Here, we asked if different ADNP mutations produce specific neuronal-like phenotypes toward better understanding and personalized medicine. (2) Methods: We employed CRISPR/Cas9 genome editing in N1E-115 neuroblastoma cells to form neuron-like cell lines expressing ADNP mutant proteins conjugated to GFP. These new cell lines were characterized by quantitative morphology, immunocytochemistry and live cell imaging. (3) Results: Our novel cell lines, constitutively expressing GFP-ADNP p.Pro403 (p.Ser404* human orthologue) and GFP-ADNP p.Tyr718* (p.Tyr719* human orthologue), revealed new and distinct phenotypes. Increased neurite numbers (day 1, in culture) and increased neurite lengths upon differentiation (day 7, in culture) were linked with p.Pro403*. In contrast, p.Tyr718* decreased cell numbers (day 1). These discrete phenotypes were associated with an increased expression of both mutant proteins in the cytoplasm. Reduced nuclear/cytoplasmic boundaries were observed in the p.Tyr718* ADNP-mutant line, with this malformation being corrected by the ADNP-derived fragment drug candidate NAP. (4) Conclusions: Distinct impairments characterize different ADNP mutants and reveal aberrant cytoplasmic-nuclear crosstalk.

## 1. Introduction

Activity-dependent neuroprotective protein (ADNP), originally discovered in our laboratory [1], contains multiple active sites [2] and is highly conserved between mice and humans at the mRNA level (90% identity) [3]. Our initial sequencing identified an ADNP homeobox domain profile (within the 1102 amino acids in human ADNP), which is known to be involved in the cell differentiation and morphogenesis [3], and it was also suggested as being implicated in DNA repair [4]. We further revealed nuclear localization and transcription factor activities of ADNP, where it regulates the chromatin structure and is implicated in the expression of hundreds of essential genes [5,6,7,8]. These findings have been verified and extended by others [9]. We subsequently identified a short neuroprotective peptide sequence, NAPVSIPQ (NAP, corresponding to the drug candidate davunetide) within the ADNP protein [1]. We further characterized NAP as a microtubule/cytoskeleton-interacting neuroprotective domain [10], which is able to replace/repair ADNP deficiencies. These previous efforts identified NAP as a peptide agent that enhances microtubule dynamics and Tau-microtubule interactions [11], protects against Tau hyper-phosphorylation [12] and tauopathy [8], and regulates actin–cytoskeletal association [2], leading to dendritic spine formation [8,10,13] that is translated to behavioral protection [13]. Conversely, ADNP deficiencies and *ADNP* gene mutations in transgenic mice led to dramatic alterations in mRNA/protein expression profiles as well as slower microtubule-dependent axonal transport, aberrant dendritic spine formation and tauopathy, which could be partially repaired by NAP treatment [7,8,12,13]. Together, these chromatin and microtubule interactions of ADNP require nuclear as well as cytoplasmic expression. Accordingly, our previous results showed specific ADNP shuttling to the cytoplasm upon neuronal differentiation [14].

Importantly, ADNP is essential for brain formation [15], with deficits in ADNP leading to autistic/intellectual disability-like and Alzheimer’s disease-like features in mice [12,13,16]. As such, de novo mutations in the *ADNP* gene lead to ADNP syndrome (also known as Helsmoortel Van Der As syndrome), which is characterized by autistic/intellectual disability [17,18,19,20]. Furthermore, we discovered an accumulation of somatic mutations in the *ADNP* gene in post-mortem Alzheimer’s disease brains and correlated this accumulation with increased severity of tauopathy [21]. In parallel, we discovered extensive tauopathy upon post-mortem analysis of a young (7-year-old) ADNP syndrome patient’s brain [22].

To specifically explore the direct effects of distinct human *ADNP* mutations on microtubule function, the p.Ser404*, p.Lys408Valfs*31, p.Tyr719*, p.Arg730*, and p.Glu830synfs*83 mutants were tested, using plasmid expression vectors and transient cellular expression. Our results revealed adverse effects on microtubule dynamics and Tau-microtubule association [2,21,22]. At the same time, NAP treatment ameliorated these microtubule-damaging effects. Interestingly, ADNP p.Lys408Valfs*31 showed a spared effect due to a regained SH3-binding motif arising due to a frameshift insertion [2]. Additionally, some *ADNP* mutations, such as those that appear in p.Arg730* (autism) and the recurrent somatic ADNP frameshift mutation p.Arg730Thrfs*4 in Alzheimer’s disease post-mortem brains, are localized to caspase cleavage sites. Indeed, ADNP residues 734–738 contain the DDSDS motif, which is a recognition motif of caspase-3 and caspase-7, suggesting direct involvement in the apoptotic pathway [23].

Further comparison of different ADNP mutants in vivo in two unique mouse models, *Adnp*^+/−^ (haploinsufficiency) [13] and CRISPR/Cas9-edited heterozygous ADNP p.Tyr718* (p.Tyr719* orthologue), revealed that the mutant allele gives rise to a gain-of-toxic-function [8]. These results are corroborated by a relatively large human cohort study of 78 children, which is suggestive of a potentially longer developmental delay in ADNP p.Tyr719* children, compared with children heterozygous for other *ADNP* mutations [20].

Lastly, RNA-seq/gene expression analyses identified converging and disparate gene expression patterns for different *ADNP* mutations [8,13,19,22], which is in partial agreement with specific DNA methylation epi-signatures, suggesting loss-of-activity coupled with gain-of-toxic-function characteristics [24]. In addition, differential ADNP cellular localization in non-neuronal cells was shown to depend on the inclusion of the nuclear localization signal in transiently expressed ADNP mutant protein species [25].

Here, we asked whether constitutively expressed diverse ADNP mutant proteins change ADNP distribution in neuronal-like cells, thereby leading to distinct effects on neuronal differentiation and survival. To answer this question, utilizing CRISPR/Cas9 genome editing, we developed novel neuroblastoma cell lines expressing different ADNP termination (stop codon) mutations under control of the native murine promoter. These strains included those expressing GFP-ADNP without mutations (used as a control), GFP-ADNP p.Pro403* (the p.Ser404* human orthologue) and GFP-ADNP p.Tyr718* (the p.Tyr719* human orthologue). Using immunocytochemistry and live cell imaging, we discovered increased neurite numbers in the neuron-like cells expressing the p.Pro403* mutant and decreased survival of cells expressing the p.Tyr718* variant. These results were associated with an increased cytoplasmatic expression of both mutant proteins. Lastly, reduced nuclear/cytoplasmic boundaries were observed in both mutant cell lines, which was a trait that was dramatically accentuated in the GFP-ADNP p.Tyr718* cell line and corrected by NAP treatment.

## 2. Materials and Methods

### 2.1. Cell Culture and Treatments

As previously described [11], mouse neuroblastoma N1E-115 cell clones (ATCC, Bethesda, MD, USA) were maintained in standard medium containing Dulbecco’s modified Eagle’s medium, 10% fetal bovine serum, 2 mM glutamine and 100 U mL^−1^ penicillin, 100 mg mL^−1^ streptomycin (Biological Industries, Beit Haemek, Israel). The cells were incubated in 95% air/5% CO_2_ in a humidified incubator at 37 °C. N1E-115 cells were differentiated into neuron-like cells upon transfer into reduced fetal bovine serum (2%) and DMSO (1.25%)-containing medium for seven days.

### 2.2. CRISPR/Cas9 DNA-Editing System

A homology-directed repair (HDR) template for generating fluorescently tagged ADNP was constructed via Gibson assembly [26]. The template structure was constructed as follows. The intron before the first coding exon of *ADNP* (5′ homology arm) was followed by *GFP* and then by the first coding exon and intron (3′ homology arm). Protospacer adjacent motif (PAM) sequences were silently altered to avoid template cleavage by Cas9. Cells were co-transfected with the PX459 vector (introducing puromycin resistance) containing active sgRNA and spCas9 and the HDR template at a 1:2 molar ratio with Jetpei transfection reagent (Polyplus transfection, New York, NY, USA). Cells were selected upon puromycin addition (8.5 µg/mL) for 12 h. After selection, the cells were allowed to recover for 48 h and further diluted (5 cells per 1 mL) and seeded in 96-well plates coated with PEI (polyethylenimine, #03880, Sigma-Aldrich, St. Louis, MO, USA) to facilitate attachment (final concentration, 0.5 cells per well). The cells were allowed to proliferate, clonal DNA was extracted, a PCR amplification of the region flanking the HDR template was performed and the product was isolated by agarose gel electrophoresis. Potential clones were sequenced (Sanger) to ensure that no other mutations had occurred. Genotyping was performed, as previously described, using standard methods [27]. We then conducted off-target screening of the most plausible off-targets according to the CRISPOR website [28], using Sanger sequencing. To introduce mutations, the same strategy was implemented for the sgRNA, although the HDR template was a single-stranded DNA (or a single-stranded donor oligonucleotides (SSODN) template of approximately 100 base pairs, ordered from Sigma-Aldrich. Sequences are provided in Supplementary Materials). The cells were transfected as above and subjected to the same enrichment, recovery and genotyping procedures. Since N1E-115 cells are polyploid, the presence of the mutants was validated by Western blotting with GFP antibodies (Santa Cruz Biotechnology, Dallas, TX, USA), as described below.

### 2.3. Western Blotting

Cell pellets were collected by centrifugation and subjected to lysis in RIPA buffer (1% NP-40, 50 mM Tris-HCl, pH 7.5, 150 mM NaCl, 0.1% SDS and 0.5% Na - deoxycholate) supplemented with protease inhibitor cocktail diluted 1:100 (Sigma-Aldrich), phosphatase inhibitor cocktail 1 (Sigma-Aldrich) and 5 mM EDTA. Cells in RIPA buffer (50–100 μL/well) were gently disrupted, mixed with a pipette, and then rotated for 20 min at 4 °C. The resulting homogenate was subjected to centrifugation (21,000× *g*, 15 min, 4 °C), and the supernatant was collected in aliquots and stored at −80 °C. Cell lysates were analyzed by sodium dodecyl sulfate (SDS)-polyacrylamide (10%) gel electrophoresis (SDS-PAGE). After separation, proteins were electro-transferred onto nitrocellulose membranes. The nitrocellulose membranes were blocked with 5% bovine serum albumin in TBST buffer including 10% TBSTX10 (50mM Tris, 150mM NaCl, Ph 7.5, adjusted with HCL) + 0.1% Tween 20 for 1 h at room temperature. The membranes were further incubated with the primary antibodies (16 h), which was followed by incubation with appropriate secondary antibodies. Secondary antibody binding was visualized with a chemiluminescence kit (Thermo Fisher Scientific, Qiryat Shemona, Israel). 

### 2.4. Antibodies Used

Anti-GFP antibodies (B-2) are mouse monoclonal IgG2as (sc-9996, Santa Cruz) recognizing amino acids 1-238, which represent full-length *Aequorea victoria* GFP. A dilution of 1:1000 was used for Western blotting. Anti-ADNP antibodies (F-9) are mouse monoclonal IgG1s (sc-376674, Santa Cruz) recognizing amino acids 1-138, mapped to the N-terminus of human ADNP [3] (dilution of 1:200). Mouse monoclonal anti-tubulin antibodies (TUB2.1), a kind gift from Professor Colin Barnstable [29], were diluted 1:100 for immunocytochemistry. The secondary antibodies used in Western blotting were horseradish peroxidase-conjugated goat anti-mouse (1:5000; Jackson, Hamburg, Germany), whereas for immunocytochemistry, goat anti-mouse Alexa Fluor 488-conjuaged antibodies (diluted 1:500) were used (Thermo Fisher Scientific).

### 2.5. IncoCyte

Cells were plated on 96-well plates (#81156, 60 μ-Dish, Ibidi, Martinsried, Germany) at a concentration of 0.3 × 10^4^ cells per well. After 24 h, the cells were washed with PBS and treated with differentiated medium containing reduced levels of fetal bovine serum (2%) and DMSO (1.25%). After 24 h of treatment, the cells were scanned with IncuCyte (×20). The number of cells and of neurites were determined per field. The image size (1408 × 1040 pixels) output from IncoCyte is defined as a field. N = 20 fields were examined for each condition analyzed.

### 2.6. Immunocytochemistry

Cells were plated on 24-well plates at a concentration of 25 × 10^4^ cells per well. Cells were immunostained with antibodies recognizing tubulin [29], and they were nuclei counter-stained/visualized with 0.2 µg/mL DAPI (Vector Lab, Burlingame, CA, USA), which was followed by incubation with the appropriate secondary antibodies. The co-localization of two flurophores is represented in white and quantified, as before [30].

### 2.7. Live Cell Imaging

Cells were plated on 35 mm dishes (#81156, 60 μ-Dish, Ibidi) at a concentration of 25 × 10^4^ cells per dish. Then, the cells were treated with differentiating medium containing reduced levels of fetal bovine serum (2%) and DMSO (1.25%) with or without NAP (10^−9^ M) or with standard medium containing NAP (10^−9^ M) or without NAP (control) for 7 days. The cells were visualized on day 1 (24 h after treatment) and on day 7 by confocal microscopy (objective × 100 (PL Apo) oil immersion, NA 1.4, Leica TCS SP8, Leica Microsystems, Wetzlar, Germany). The number of cells that contained diffuse nuclear/cytoplasmic GFP-ADNP distribution with indiscernible nuclear boundaries (as identified by GFP fluorescent intensity) was counted and divided by the total number of cells in the field.

### 2.8. Statistical Analysis

Data are presented as the mean ± S.E.M. from at least two/three independent experiments. Statistical analysis of the data was performed by one-/two-way ANOVA (followed by a Tukey’s post hoc test) using PRISM Statistics software, version 24 (IBM, Armonk, NY, USA). * *p* < 0.05, ** *p* < 0.01, *** *p* < 0.001.

## 3. Results

### 3.1. Genome Editing to Generate Cell Lines That Express GFP-ADNP, GFP-ADNP p.403* and GFP-ADNP p.Tyr718*

**Figure 1:**
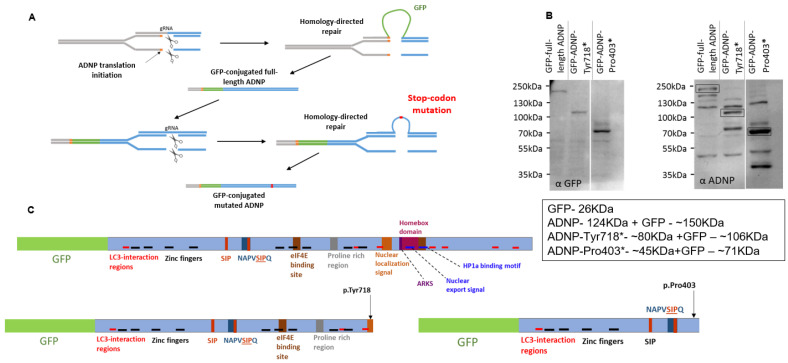
Cell model genome editing and validation. (**A**) Schematic representation of the CRISPR/Cas9 DNA-editing system used to establish neuroblastoma cell lines expressing full-length ADNP conjugated to GFP and truncated ADNP proteins. (**B**) Immunoblotting with anti-GFP (**left panel**) and anti-ADNP (**right panel**) antibodies was performed to confirm the expression of GFP-tagged proteins. The α-GFP panel depicts only GFP-conjugated proteins, while the α-ADNP panel depicts all ADNP immunoreactive forms. (**C**) Schematic representation of functional protein regions depicted along full-length ADNP and both mutants, adapted from our latest publication, which further identified SH3 domains, including NAPVSIP in the NAP sequence and an actin-binding site [2].

Figure 1A presents a schematic representation of the CRISPR/Cas9 DNA-editing paradigm used that resulted in the creation of murine neuroblastoma cell lines expressing full-length ADNP conjugated to GFP or mutant (truncated) ADNP proteins. Western blot analysis with anti-GFP (Figure 1B, left panel) and anti-ADNP (Figure 1B, right panel, bands marked by rectangles) antibodies precisely identified full-length ADNP and ADNP mutant proteins conjugated to GFP. Additional ADNP-immunoreactive bands were seen using anti-ADNP antibodies, possibly corresponding to full-length ADNP lacking the GFP-tag, reduced antibody specificity and/or ADNP presenting additional post-translational modifications.

### 3.2. GFP-ADNP p.Pro403* Increases Neurite Numbers, While GFP-ADNP p.Tyr718* Produces Cell Death

**Figure 2:**
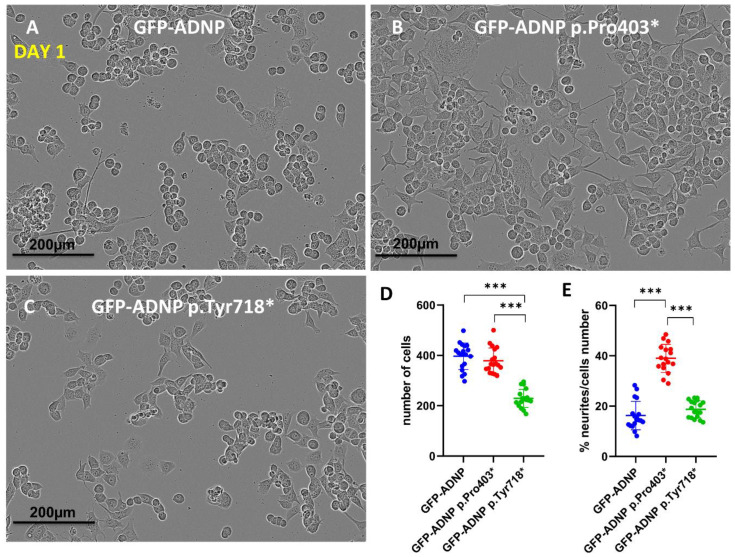
IncoCyte images and analysis indicate differential effects of selected ADNP mutants. (**A**) Neuroblastoma cell lines expressing full-length ADNP or (**B**,**C**) truncated ADNP proteins were scanned with IncoCyte (×20) following a 24 h incubation in differentiation medium. Image size (1408 × 1040 pixels), represents one field, with 20 fields being analyzed for each condition. (**D**) When the number of counted cells per field (N = 20) was analyzed statistically, significant cell death in the GFP-ADNP p.Tyr718*-expressing line was revealed, *** *p* < 0.001. (**E**) Calculating the percentage of neurites in each field showed increased neurite levels in cells expressing GFP-ADNP p.Pro403*.

Confocal images portraying GFP-ADNP, GFP-ADNP Pro403 and GFP-ADNP Tyr718 expression (Figure 2A–C), together with cell and neurite counting (Figure 2D,E), identified a 50% reduction in cell numbers 24 h after plating in the case of the GFP-ADNP p.Tyr718*-expressing cell line, in comparison to GFP-ADNP- (ADNP without mutation used as control) and GFP-ADNP p.Pro403*-expressing cells (Figure 2A–D). In contrast, the GFP-ADNP p.Pro403*-expressing line showed a 50% increase in the number of neurites per cell relative to the GFP-ADNP- and GFP-ADNP p.Pro403*-expressing lines (Figure 2A–C,E). These results suggest different impacts of the two mutants during early development.

### 3.3. Dramatically Increased Cytoplasmic Distribution of GFP-ADNP p.403* and GFP-ADNP p.Tyr718* in Comparison to GFP-ADNP during Neuronal-like Differentiation

**Figure 3:**
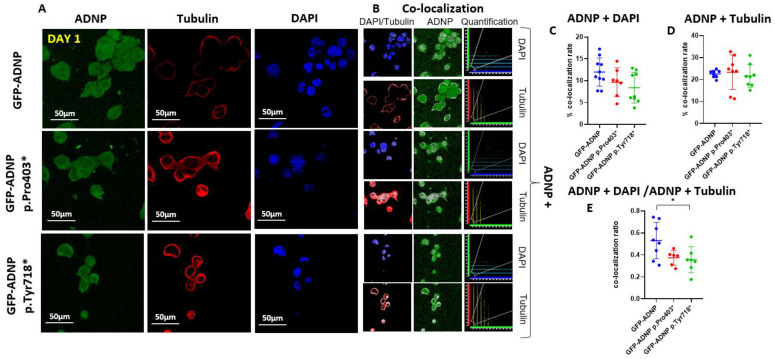
Immunostaining of tubulin and staining with DAPI after 24 h in culture revealed a minor effect of mutant protein expression. (**A**) Neuroblastoma cell lines expressing full-length ADNP or truncated ADNP proteins conjugated to GFP (**green**) were immunostained with monoclonal anti-tubulin (**red**) antibodies (TUB2.1), while cell nuclei were stained/visualized with DAPI (DNA, **blue**). (**B**) Nuclear or cytoplasmic co-localization of the DAPI and ADNP or tubulin and ADNP, respectively, is represented by the white color (apparent dots); ×63 oil immersion lens. The quantification graphs show scatterplots of the white dots, depicting red (tubulin) or blue (DAPI) + green (ADNP) turned into white pixel intensities of the image shown in the adjacent pictures. (**C**) Quantitative analysis of ADNP–DAPI co-localization. (**D**) Quantitative analysis of ADNP-tubulin co-localization. (**E**) Quantitative analysis of ADNP–DAPI/ADNP-tubulin ratio of staining-based co-localization is represented, * *p* < 0.05. Nine fields were analyzed for each condition. A field is defined as one image by the confocal microscope (one image size = 1024 × 1024 pixels). Apparently, the majority of ADNP did not associate with microtubules or DNA (DAPI, **D**,**E**), possibly reflecting the multiple protein interaction sites on the protein (Figure 1C).

**Figure 4:**
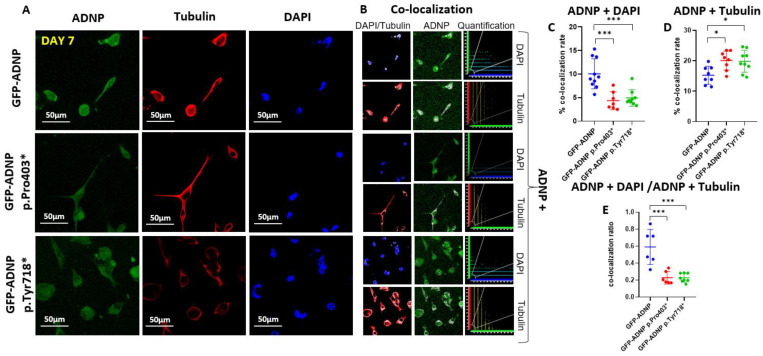
Immunostaining of tubulin and staining with DAPI in differentiated neuroblastoma-like neuronal cells (day 7 in culture) indicated an increased cytoplasmic localization of the ADNP mutants. (**A**) Neuroblastoma cell lines expressing full-length ADNP or truncated ADNP proteins conjugated to GFP (**green**) were immunostained with anti-tubulin (**red**) monoclonal antibodies (TUB2.1), while cell nuclei were stained/visualized with DAPI (**blue**). (**B**) Nuclear or cytoplasmic co-localization of the DAPI and ADNP or tubulin and ADNP, respectively, is represented by the white color; ×63 oil immersion lens. For further explanations, please see Figure 3B. (**C**) Quantitative analysis of ADNP–DAPI co-localization. (**D**) Quantitative analysis of ADNP-tubulin co-localization. (**E**) Quantitative analysis of the ADNP–DAPI/ADNP-tubulin ratio, reflected by merged staining upon co-localization is presented. Nine images were quantified, as described in the legend to Figure 3. Apparently, the majority of ADNP did not associate with microtubules or DNA, possibly reflecting the multiple protein interaction sites on the protein (Figure 1C). Asterisks denote: * *p* < 0.05; *** *p* < 0.001.

To precisely identify ADNP and mutant ADNP cellular distribution, we performed immunocytochemistry of fixed cells after day 1 (Figure 3A) and day 7 (Figure 4A) of differentiation using monoclonal anti-tubulin antibodies (TUB2.1) [29] and DAPI for DNA staining. ADNP proteins were identified due to their conjugation to GFP (Figure 3A and Figure 4A). The white color represents the co-localization (Figure 3B and Figure 4B) of ADNP with microtubules (tubulin) or nuclear localization (DAPI DNA staining). Quantification of the immunocytochemical results from cells fixed after 24 h in culture (Day 1; Figure 3C–E) showed almost no differences, except for a small yet significant reduction in nuclear/cytoplasmatic distribution (ADNP + DAPI/ADNP + Tubulin) of GFP-ADNP p.718* in comparison to GFP-ADNP (Figure 3E). In contrast, after complete neuronal-like differentiation (Day 7; Figure 4), there was a three-fold reduction in nuclear co-localization of the mutated proteins as compared to the GFP-ADNP control (Figure 4C). This decrease in nuclear localization was coupled with an increase in cytoplasmic (tubulin-associated) distribution of the mutants (Figure 4D), which together amounted to a dramatic five-fold change in the nuclear/cytoplasmatic distribution of both mutant forms of ADNP as compared with the GFP-ADNP chimera (Figure 4E).

### 3.4. GFP-ADNP p.Pro403* Increases Neurite Length Whereas GFP-ADNP p.Tyr718* Produces Visually Ambiguous Nuclear Envelope/Cytoplasmic Boundaries in Neuron-like Differentiated Cells

**Figure 5:**
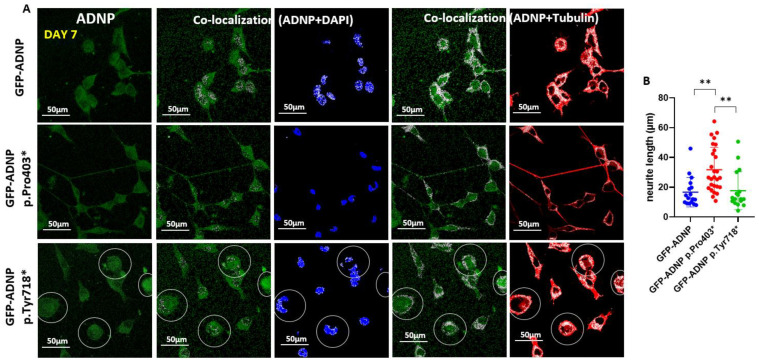
Close inspection of tubulin and DAPI in differentiated neuronal-like cells (day 7 in culture) indicates increased neurite length upon GFP-ADNP p.Pro403* mutant expression. (**A**) Images show four-fold enlarged co-localization regions of Figure 4A,B. Cells with diffusely distributed stained proteins, identified by diffuse nuclear/cytoplasmic co-localization, are circled. (**B**) Neurite lengths were measured using Fiji-ImageJ image processing software [31]. The longest neurites were measured in GFP-ADNP p.Pro403*-expressing cells, ** *p* < 0.01.

Closer inspection of the immunostained cells (Figure 5A, magnifying the size of the co-localization panels from Figure 4) identified increased neurite length in the GFP-ADNP p.Pro403*-expressing differentiated cells in comparison to the GFP-ADNP-expressing cells (Figure 5A). Interestingly, cells expressing GFP-ADNP p.Tyr718* exhibited a somewhat undiscernible nuclear envelope, making it difficult to discern nuclear vs. cytoplasmic distribution (Figure 5A, circled representative cells).

**Figure 6:**
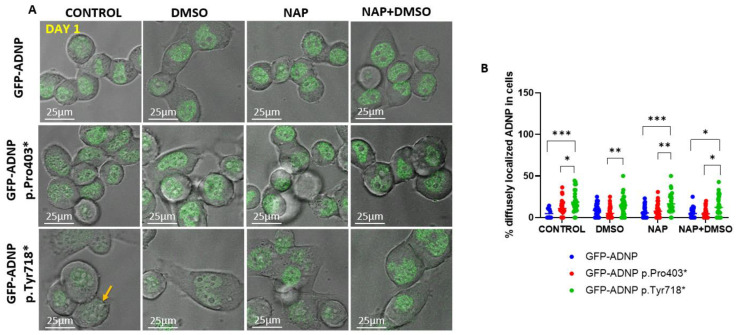
Confocal live imaging of non-differentiated cells (day 1 in culture) showing the distinct cellular distribution of GFP-ADNP p.Tyr719*. (**A**) Representative confocal/fluorescent merged images (Leica SP8) after one day in culture are shown. Selected cells showing diffusely localized GFP-ADNP p.Tyr718* are indicated with yellow arrows, which is indicative of cells with indiscernible nuclear/cytoplasmic boundaries. (**B**) Thirty-four images (1024 × 1024 pixels/image) were analyzed, and the percentage of diffusely localized ADNP outside the nucleus (no nuclear boundaries) was calculated. CONTROL = non-differentiating medium; DMSO = differentiating medium (reduced serum + DMSO); NAP = non-differentiating medium + NAP; NAP + DMSO = differentiating medium + NAP, * *p* < 0.05; ** *p* < 0.01; *** *p* < 0.001.

**Figure 7:**
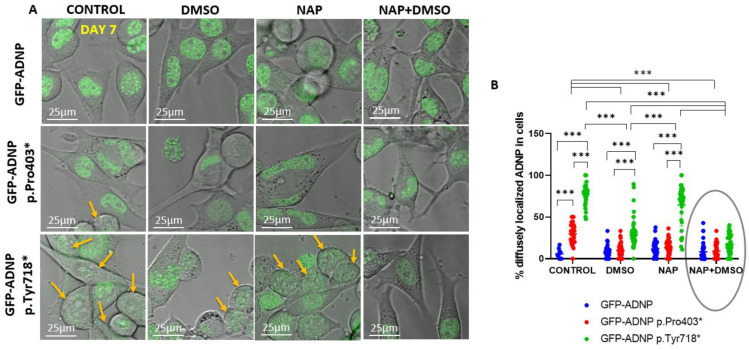
Confocal live cell imaging of neuronal-like differentiated and non-differentiated cells expressing full-length ADNP or truncated ADNP conjugated to GFP, indicating correction of mutant-induced undiscernible nuclear envelopes. (**A**,**B**) Experiments were conducted as detailed in Figure 6; however, here, the cells used were incubated for 7 days in culture to reach full differentiation. Yellow arrows indicate diffusely distributed mutant ADNP in cells showing indiscernible nuclear/cytoplasmic boundaries. The added ellipse indicates NAP-mediated protection, *** *p* < 0.001.

To quantify GFP-ADNP p.Tyr718* cellular diffuse nuclear/cytoplasmic distribution, considering how its expression made it difficult to identify nuclear boundaries, we turned to live cell imaging in differentiated and non-differentiated cell lines. On day 1 (24 h post-plating, Figure 6A), there was a minor but statistically significant difference in the diffuse nuclear/cytoplasmic distribution of GFP-ADNP p.Tyr718* in comparison to GFP-ADNP or GFP-ADNP Pro403* (Figure 6B). These differences were much greater after 7 days in culture in non-differentiating cells (without DMSO + serum deprivation) (Figure 7A,B) and affected both GFP-ADNP mutant-expressing cell lines. DMSO + serum reduction-induced differentiation or NAP treatment corrected this phenotype in GFP-ADNP Pro403* cells, whereas co-incubation with NAP and differentiation medium was required for the complete restoration of GFP-ADNP p.Tyr718* nuclear distribution (Figure 7B).

## 4. Discussion

Using CRISPR/Cas9 genome editing, we developed, for the first time, novel neuroblastoma cell lines that constitutively expressed GFP-ADNP p.Pro403* (the p.Ser404* human orthologue) and GFP-ADNP p.Tyr718* (the p.Tyr719* human orthologue) under their endogenous murine promoters. Then, using quantitative cellular morphology analysis, immunocytochemistry, and live cell imaging, we revealed new phenotypes associated with specific *ADNP* mutations. Specifically, increased neurite numbers and neurite lengths were linked with the p.Pro403* mutant early (1 day) and late (7 days) into neuronal-like differentiation, respectively. In contrast, decreased cellular survival was linked with the ADNP p.Tyr718* mutant. These findings were coupled with an increased cytoplasmic expression of both mutant proteins and with reduced nuclear/cytoplasmic boundaries, which were dramatically accentuated for the ADNP p.Tyr718* mutant cell line and corrected by NAP treatment in differentiated neuronal-like cells.

Placing our current findings in the context of the existing literature, we originally discovered a nuclear localization site in the ADNP protein sequence [3], with this sequence being specifically disrupted in the p.Tyr718* variant. Interestingly, comparing our two mouse models, i.e., *Adnp*^+/−^ and genome-edited ADNP p.Tyr718* (Tyr)-expressing mice, we discovered that the p.Tyr718* mutation is associated with gain-of-toxic-function and early post-natal death [8], which is in agreement with the current findings. Furthermore, previous natural clinical history studies suggested that the orthologue human p.Tyr719* mutation causes a more severe developmental impairment (motor and sensory), as compared to other *ADNP* mutations [20].

The surprising dichotomous discovery of increased neurite outgrowth, a specific gain-of-toxic-function associated with the p.Pro403* mutation in ADNP, contrasting the cell survival deleterious effect of the p.Tyr718* mutation, may be related to the two distinct and partially opposing genomic DNA methylation epi-signatures in ADNP syndrome blood. The “epi-ADNP-1” epi-signature that includes ≈6000 mostly hypo-methylated CpGs correlates with mutations in the N- and C-terminal (e.g., p.Ser404*, human orthologue) regions of ADNP, while the “epi-ADNP-2” epi-signature includes ≈1000 predominantly hyper-methylated CpGs, with epi-ADNP-2 mutations being centered on the nuclear localization signal (e.g., p.Tyr719*, human orthologue) [24,32]. With this in mind, closely inspecting our results on early tooth eruption in 54 children shows that most of the children who did not have early tooth eruption presented the epi-ADNP-2 signature [19], thereby connecting epigenetics to phenotypic expression. Furthermore, the observed phenotype of accumulated ADNP p.Tyr718* adjacent to the nuclear envelope, the site of heterochromatin concentration [33,34], albeit with indiscernible nuclear boundaries, as well as the dispersal of ADNP p.Pro403* throughout the nucleus, corroborates the “epi-ADNP-2” pattern of increased heterochromatin distribution resulting from ADNP p.Tyr718* expression and the “epi-ADNP-1” pattern of increased euchromatin distribution due to ADNP p.Pro403* expression, respectively.

The neurite outgrowth-promoting effect of ADNP is linked to both cytoplasmic/microtubule interactions and the proven impact on microtubule dynamics, microtubule–Tau interaction [11] and axonal transport [7], as well as binding to the nuclear SWI/SNF chromatin remodeling complex [5,35], impacting neural differentiation. As such, this suggests a delicate balance of control. In this respect, ADNP auto-regulates its own promoter [6,36], such that the different mutants may cause temporal/transient changes in heterozygous ADNP expression, affecting phenotypic outcomes. Interestingly, increased sprouting was previously linked with autistic characteristics [37], with these differential mutational effects of ADNP possibly partly explaining the impact of heterogeneous mutation on the ADNP syndrome autistic phenotype, although not all ADNP children were strictly assigned a diagnosis of autism [20]. On the other hand, decreases in ADNP levels have been linked with diminished dendritic spines and synaptic plasticity in mice [13,38] and cognitive deficiencies in mice [13,38] and humans, with most if not all ADNP syndrome patients suffering from intellectual disabilities [18,19,20,39].

A most intriguing finding of the current study is the apparent fading of nuclear boundaries upon the expression of both ADNP mutants, with the effect being most pronounced with the p.Tyr718* mutant. A direct connection of ADNP to nuclear lamina integrity is the known binding of ADNP to heterochromatin protein 1 (HP1) [6,40,41,42], specifically, HP1 alpha [6], an integral part of the nuclear lamina [43]. The ADNP–HP1 interaction site is lost in both of the currently tested mutants, partly explaining the apparent disrupted nuclear lamina seen prior to differentiation in cells expressing either variant. Indeed, as indicated above, RNA-seq comparing different ADNP mutations identified shared transcriptional outcomes [19,22], although distinct mechanisms were apparent as well. Unique regulated genes shared by p.Tyr719* humans and p.Tyr718* mice are stomatin (STOM) and DnaJ heat shock protein family (Hsp40) member B4 (DNAJB4). Importantly, NAP restored the expression patterns these two gene transcripts in the Tyr718* (Tyr) mice [8]. Stomatin is an integral membrane protein associating with actin [44] (with ADNP associating with actin, as well [2]), while DNAJB4 belongs to the 40 kDa family of chaperone proteins, which are important for nuclear import [45], and dysregulation of these genes may also be involved in the currently observed phenotype. The ADNP-mediated effect on the nuclear envelope phenotype is further suggested to be linked to NAP-mediated regulation of gene expression/function with a strong emphasis on healthy aging-associated genes, such as sirtuin 1 (SIRT1) [30] and Forkhead Box O3 (FOXO3) [8], and with nuclear lamina integrity, which is intimately involved with the regulation of aging [46].

Lastly, it is worth discussing the possibility that cellular toxicity resulting from the expression of ADNP p.Tyr718* was due to a non-nuclear mechanism. Thus, our findings of increased cell death in cells expressing GFP fused to ADNP p.Tyr718* may be associated with a potential triggering of progressive apoptotic or necrotic cell death. In its early stages, this cell death would manifest with caspase-dependent and caspase-independent breakdown of the nuclear envelope [47], which is in line with our current observations, and with NAP protecting against caspase activation and apoptosis [23,48,49,50].

Future studies aimed at translating the current cellular and previous animal studies to clinical practice [51] for better understanding of mutational differences should be undertaken, starting with a clinical trial in ADNP syndrome children, harnessing the NAP (davunetide) enhancement/replacement therapy of deficient ADNP.

## 5. Conclusions

Distinct cellular phenotypes characterize different ADNP mutants and explain the more severe phenotype of the p.Tyr718*/719* mutant. Understanding the versatile phenotypes coupled with the ameliorative effects of NAP (davunetide) on shared phenotypes accentuated in the prevalent p.Tyr718* mutation provides hope for future therapeutic interventions.

## 6. Patents

NAP (CP201, davunetide) use is under patent protection (US patent nos. US7960334, US8618043, and USWO2017130190A1) (I.G.), PCT/IL2020/051010 (I.G.) and PCT applications (I.G., inventor and Y.I.-P., M.G., and G.K., contributing scientists).

## Data Availability

Not applicable.

## Supplementary Materials

The following supporting information can be downloaded at: https://www.mdpi.com/article/10.3390/cells11192994/s1, CRISPR/Cas9 DNA-editing.
