# Supplementary material for: Distinct Impairments Characterizing Different ADNP Mutants Reveal Aberrant Cytoplasmic-Nuclear Crosstalk
Source: Cells. 2022 Sep 26;11(19):2994. doi: 10.3390/cells11192994 (PMC9563912; doi:10.3390/cells11192994)
Supplement: Supplementary file 1 [file cells-11-02994-s001.zip › cells-1925124-supplementary.pdf]

Supplementary Materials:

CRISPR/Cas9 DNA-editing

DNA sequence of the template for tagging ADNP with GFP (small script denotes changes made in the sgRNA sequence to avoid template degradation):

AAGTGCACGAATGTTCACAGGGttttgtttttttttttttAAAGGATCCCAGAA
AGACAGGATTCACTGTATGTTCTGATGCCACAAACATGCTTTTTCCC
ATCTATGTGTTTATTCTGTGGCCTATGTGAGGCCTTTGCCCTGATGTG
TTTCCACCCAGGGAGCCAGTCCCACCACCATTTGGGACTTTGGTTTG
AGGAGGAGACCGAAACAGTCACTGAATGACATGTGGCTTCTGTGAG
TGTCATGGCCTTGATCAGGACATTTCAGATGGCAGAATCCTCCCCTC
AGCACTGAAAGCTGAGTGATGCTGTGAAGCTCGGAGTGGCTCCAGC
TTCGTAGGTCCAGCTTCATCGAGAGGAGACATGCGCACTCGATTTGG
CTTTGAGATAGCTGGGTGATATTGTTGCCCTCATTTTCCAACCTCAGG
TTCATCTCTGAACAGATTTTTACAGCTTGCTGAGACTTGTGTGCCTCG
ACTTGTGGTCTAAAATATCCTTTTTTGAAATAGGGAAGGAAGGAAG
GCTAACAAGCACTCACCTATTTGCCTGCTACTCTTAAGTGTTCCATAC
TAGGCGAGGAATTCTCAACTGAtaGAGCtAtaGTTTGGGATCCTATCAT
CAGTcaAATTAGTAACTTTTACAAGTTGTACATGTTTTGACTTTGACTT
AAAtttttttttCCAATAGAAACTATGGTGAGCAAGGGCGAGGAGCTGTT
CACCGGGGTGGTGCCCATCCTGGTCGAGCTGGACGGCGACGTAAAC
GGCCACAAGTTCAGCGTGTCCGGCGAGGGCGAGGGCGATGCCACCT
ACGGCAAGCTGACCCTGAAGTTCATCTGCACCACCGGCAAGCTGCC
CGTGCCCTGGCCCACCCTCGTGACCACCCTGACCTACGGCGTGCAGT
GCTTCAGCCGCTACCCCGACCACATGAAGCAGCACGACTTCTTCAA
GTCCGCCATGCCCGAAGGCTACGTCCAGGAGCGCACCATCTTCTTCA
AGGACGACGGCAACTACAAGACCCGCGCCGAGGTGAAGTTCGAGG
GCGACACCCTGGTGAACCGCATCGAGCTGAAGGGCATCGACTTCAA
GGAGGACGGCAACATCCTGGGGCACAAGCTGGAGTACAACTACAA
CAGCCACAACGTCTATATCATGGCCGACAAGCAGAAGAACGGCATC
AAGGTGAACTTCAAGATCCGCCACAACATCGAGGACGGCAGCGTGC
AGCTCGCCGACCACTACCAGCAGAACACCCCCATCGGCGACGGCCC
CGTGCTGCTGCCCGACAACCACTACCTGAGCACCCAGTCCGCCCTG
AGCAAAGACCCCAACGAGAAGCGCGATCACATGGTCCTGCTGGAGT
TCGTGACCGCCGCCGGGATCACTCTCGGCATGGACGAGCTGTACAA
GATGTTCCAACTTCCTGTCAACAATCTTGGCAGTTTAAGAAAAGCCC
GGAAAACTGTGAAAAAAATACTTAGTGACATTGGGTTaGAATACTGT
AAAGAACATATAGAAGTAAGTAGCATGCCATTTTTAAATTTTAATGT
GACTCAGCTCCTGATCCATTACTGTGTCCATGGGCAAAGATGGTTCA
CTAATAGGTGTGGTAGTAAATTTTAACTAGACCTTCAGCTTCACCAA
AACCTTCTCACGTAGGATAGCTTACTTCATTTTCCCTGAAAGCCTGTT
CATCTTGGTAACCTGAGGCAAAACTAATTACAGCACAGAGAATGTA
CACAAGTCAGACAGTGCACATGGTGCTTTAAAACTGTCTATGAGCTT
TCTCCTTGAAATAACCTTTAGGGGCTCTAGGAATTCTTGTCTCCAGTC

TATGAAAGAAGAATTGGGAAAGCAGAGATGCCAGGCTCTCAGCTA
GGGAAGGGGCAGTGAGGACTGTAGGGCCTGTTCTCCTTCTGATAGG
CCTGGGTGTTTCTGTTGACCTGTTGAGGCACAGTCGTCTGAGTGTTTG
TCATCTCTTTCCTTAGGATTTGAGCACTTGTCAGCCTTCTGAACTATC
AAAGACTTATCTTGCCCTTGTGTAACAGTTTGGCCTTTCAGACTGTTT
CCTATCCTTTTTATTCTGAGCCTTTCAAGCCCCATAAGCTAGATAGAG
ACGTCTCCTCCTTTTCCAGATCTCCTTGTTCCCAATCTTACAGTTAGTT
AGACACAACCTCCCATTGAAGTGCTTACAtctaga

Adding GFP sgRNA sequences (small script denotes changes made in the sgRNA sequence to avoid secondary structures):

GTTTGGGATCCTATCATCAG
GGCGAGGAATTCTCAACTGA
taGTTTGGGATCCTATCATC

SSODN sequence of the template editing the Tyr718 mutation (small script denotes changes made in the sgRNA sequence to avoid template degradation and adding stop codon):

GACAAGACAAACGCACCTTCTCGGCTCAATCA
TCTCCAGGCCTGGCgCCTGTcAAGCGCAtTAaGAGCAaATGGAGTTTC
CACTGCTAAAAAAGC

Tyr718 sgRNA sequences:

GTGAAGCGCACGTATGAGCA
TGTGAAGCGCACGTATGAGC
GAAGCGCACGTATGAGCAGA

SSODN DNA sequence of the template editing the Pro403 mutation (small script denotes changes made in the sgRNA sequence to avoid template degradation and adding stop codon):

GCAGCAGCCAGGTACTCCCTGCAGACTGCCAACACCTCTCTACCttaA
GGCCAAGTGAAGTCTCCCTCTGTGTCTCAGTCACAGGCATCTAGAGT
ATTA

Pro403 sgRNA sequences:

GCCAACACCTCTCTACCCCC
CAGGCCAAGTGAAGTCTCCC
CCAGGCCAAGTGAAGTCTCC

Single guide RNA (sgRNA), single-stranded oligodeoxynucleotide (SSODN)
